# Supplementary material for: Postnatal Cytoarchitecture and Neurochemical Hippocampal Dysfunction in Down Syndrome
Source: J Clin Med. 2021 Jul 31;10(15):3414. doi: 10.3390/jcm10153414 (PMC8347520; doi:10.3390/jcm10153414)
Supplement: Supplementary file 1 [file jcm-10-03414-s001.zip › jcm-1277465-Supplemental Material.pdf]

Supplemental Figures

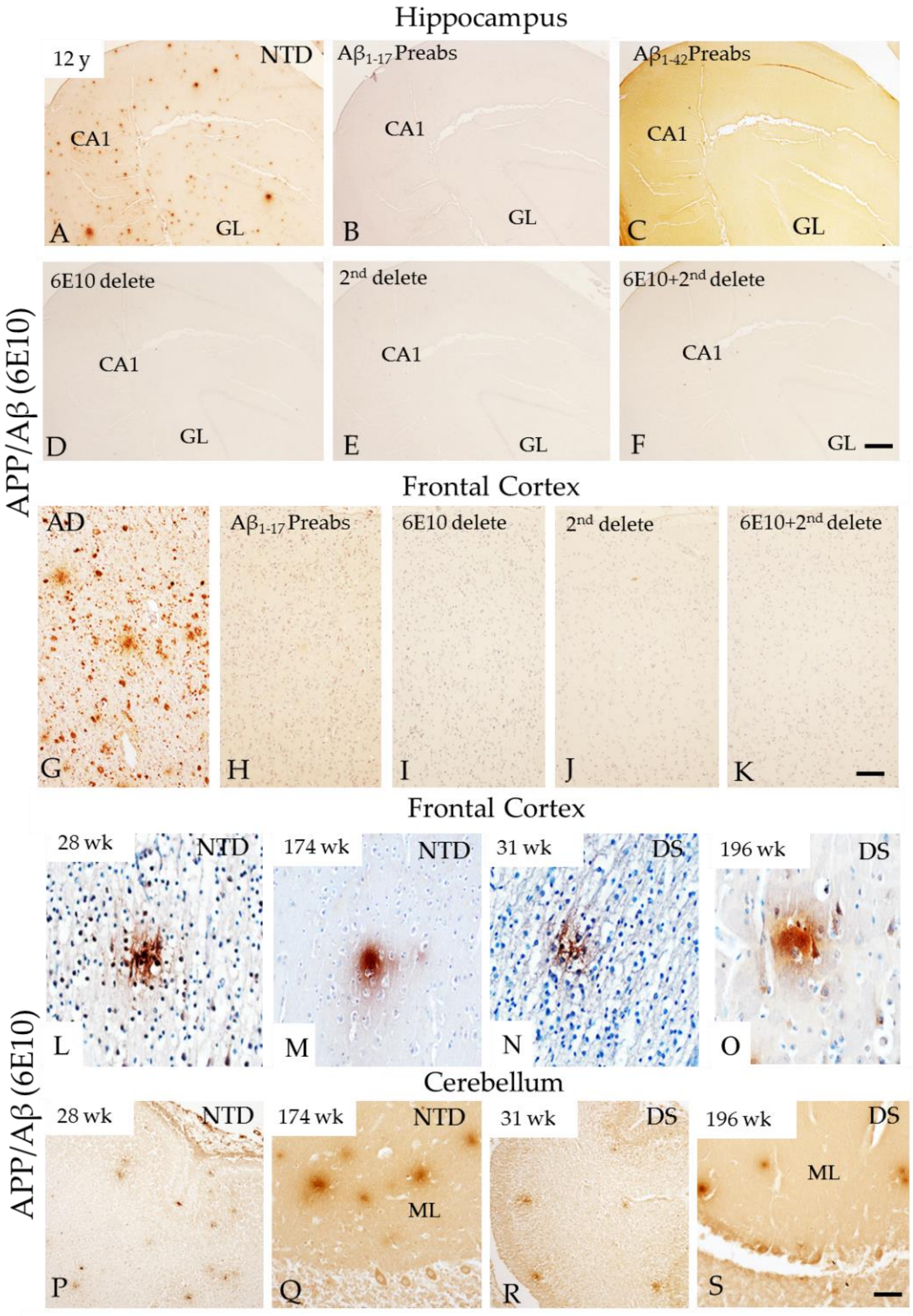

Figure S1. Specificity of APP/Aβ immunoreaction in the postnatal hippocampus. Low-power images of a NTD 12-year-old hippocampus (**A-F**) and an AD 82-year-old female showing APP/Aβ-ir deposits (**A, G**) as well as the lack of APP/Aβ immunoreactivity following preabsorption of 6E10 with $A\beta_{1-17}$ (**B, H**) and $A\beta_{1-42}$ (**C**) peptides and after primary (**D, H**), secondary (**E, J**) and primary+secondary (**F, K**) deletion experiments. Postnatal frontal and (**L-O**) cerebellum (**P-S**) images showing APP/Aβ-ir like-plaques/burst profiles at 28 and 174 in NTD (**L, M, P, Q**) and in DS 31 and 196 postnatal weeks (**N, O, R, S**). Tissue in panels **B**, **D-O** was counterstained with hematoxylin. Abbreviations: $A\beta_{1-17}$ Preabs=preabsorption with $A\beta_{1-17}$ peptide, $A\beta_{1-42}$ Preabs=preabsorption with $A\beta_{1-42}$ peptide, CA1=hippocampal subfield CA1, GL=granule cell layer, ML=molecular layer of the cerebellum. Scale bars: **F**=500 μm applies **A-E, K**=100 μm applies **G-J**, **P** and **R**, **S**=50 μm applies **Q**.

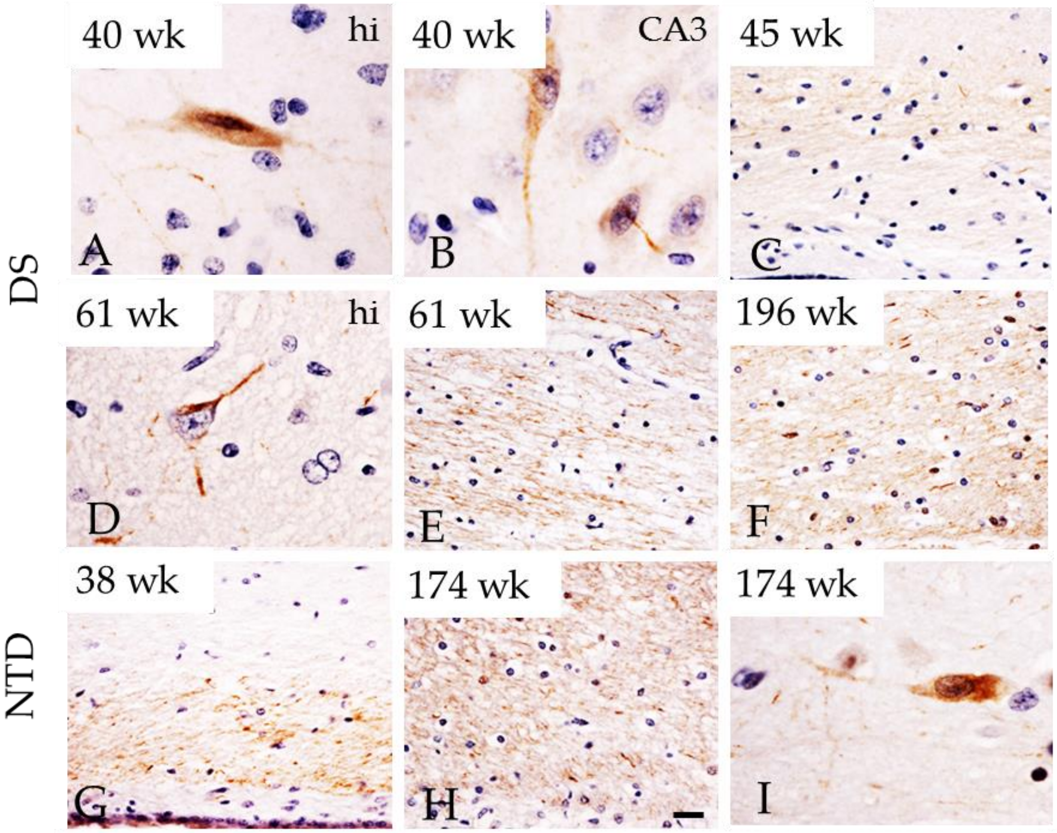

Figure S2. SMI-34 immunostaining in postnatal DS and NTD hippocampus. SMI-34-ir cells in the hi and CA3 field in a 40 (**A-F**) and a 61 week DS case, as well as positive fibers in the alvear tract in a 45, 61 and 196 week DS case. SMI-34-ir alvear fibers were observed in a 38 week-old NTD case and both alvear positive fibers and cells in a 174 week NTD case. Note many more SMI-34-ir fibers in the alvear tract in the oldest DS and NTD cases. Abbreviations: CA3=hippocampal subfield CA3, hi=hilus. Scale bar in **H**=25 μm for **C, E-H** and 10 μm in **A, B, D, I**.

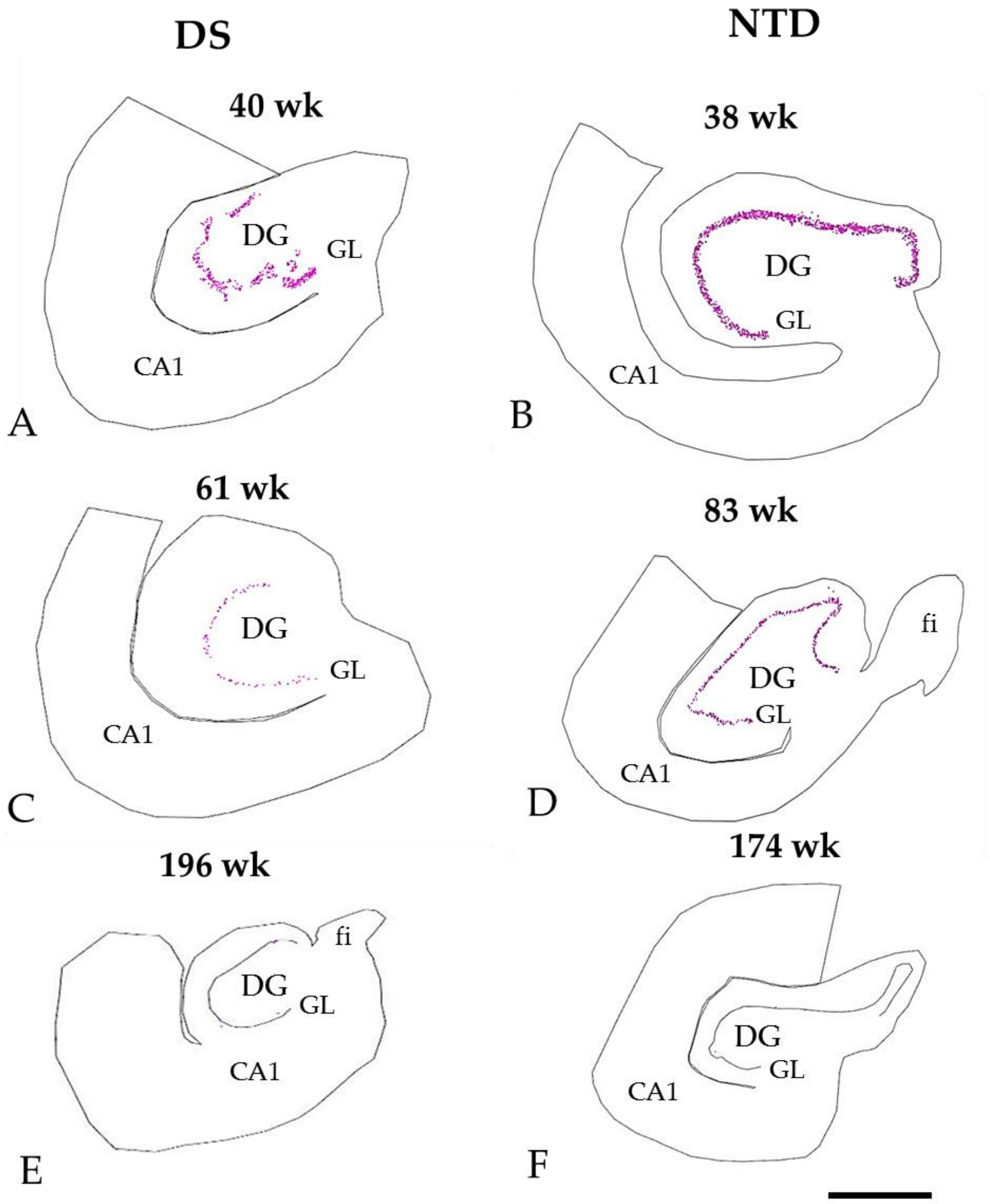

Figure S3. Distribution of DCX positive granule cells in the postnatal DG in DS and NTD. Hippocampal tracings showing distribution of DCX positive granule cells in the DG at postnatal weeks 40 (**A**), 61 (**C**), 196 (**E**) in DS and at 38 (**B**), 83 (**D**), and 174 (**F**) weeks in NTD cases. Note the age-related reduction in the number of DCX positive cells in both groups. Less cells were observed in the 61 week-old (**C**) DS case compared to the 83 week-old (**D**) NTD case. Tracings were performed with a Neurolucida Tracing Software at 10x (MBF Bioscience Inc.). Abbreviations: CA1=hippocampal subfield CA1, DG=dentate gyrus, fi=hippocampal fimbria, GL=granule cell layer, hi=hilus. Scale bar = 1.25 mm.

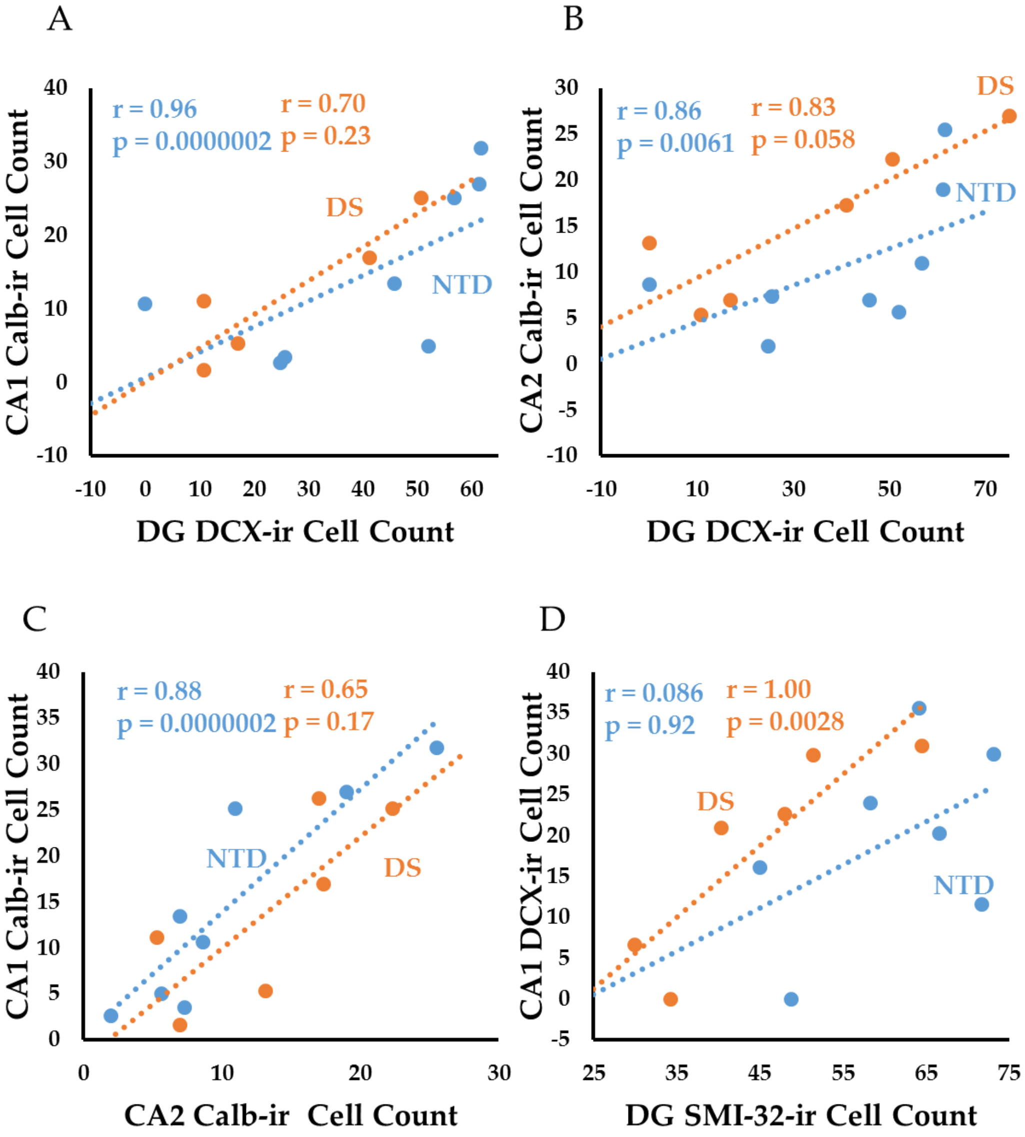

Figure S4. Correlations between postnatal hippocampal cell counts in DS and NTD. Linear regression analysis showing positive correlations between DG DCX-ir cell counts and CA1 Calb (**A**; Spearman rank, $r = 0.96$ $p = 0.0000002$) and CA2 Calb (**B**; Spearman rank, $r = 0.85$ $p = 0.00609$) positive counts as well as CA1 Calb-ir cell counts and CA2 Calb-ir cell counts (**C**; Spearman rank, $r = 0.88$ $p = 0.0000002$) in NTD, while in DS CA1 DCX-ir counts correlated with DG SMI-32 (**D**; Spearman rank, $r = 1.00$ $p = 0.0027$).

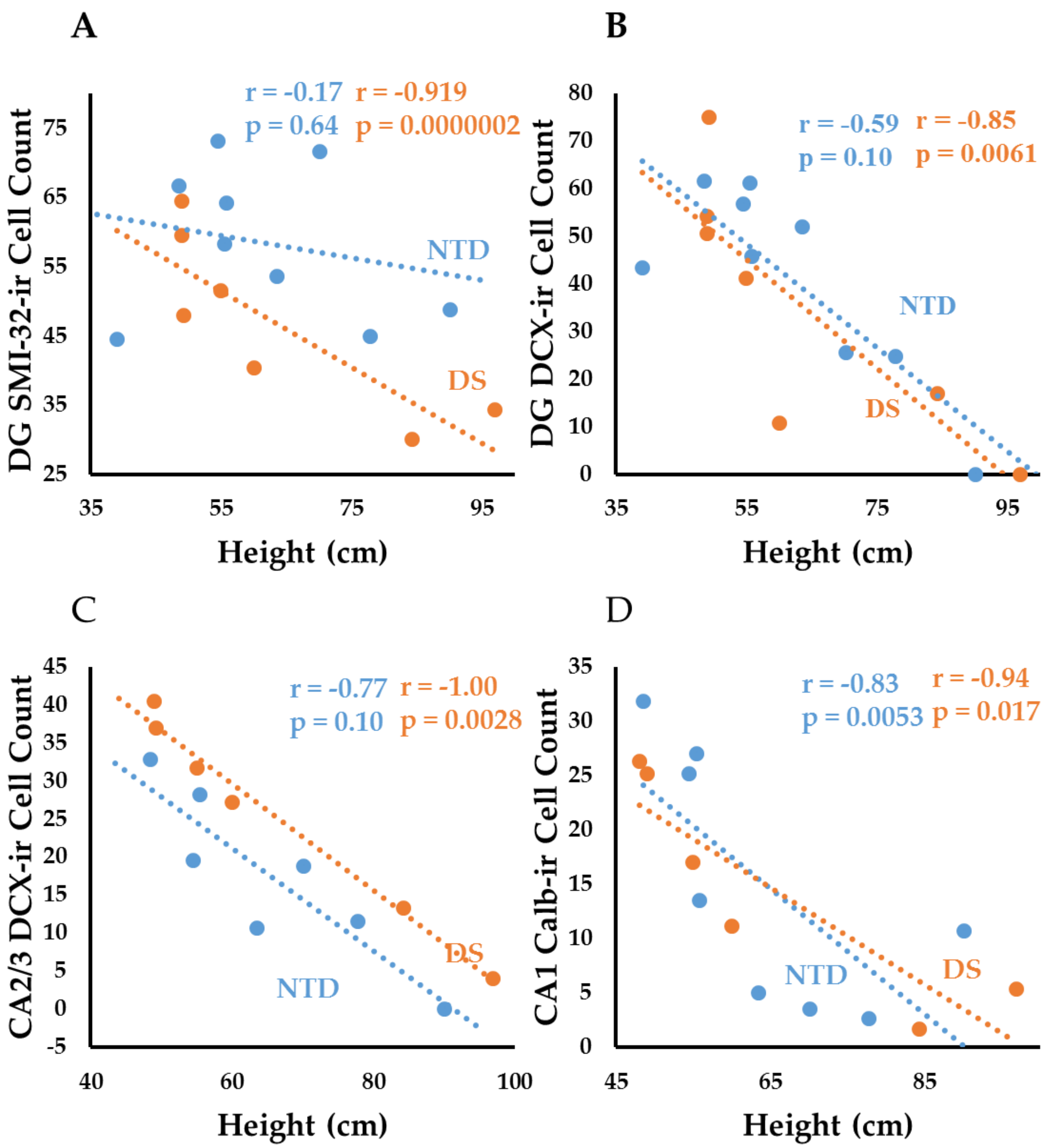

Figure S5. Correlations between postnatal hippocampal cell counts and height in DS and NTD. Linear regression analysis showing negative correlations between height and DG SMI-32 (**A**; Spearman rank, $r = -0.86$; $p = 0.0061$), DG DCX (**B**; Spearman rank, $r = -0.85$ $p = 0.0061$), and CA2/3 DCX (**C**; Spearman rank, $r = -1.0$ $p = 0.0028$) in DS, and Calb-ir CA1 (**D**; Spearman rank, $r = -0.83$ $p = 0.0053$) counts in NTD.

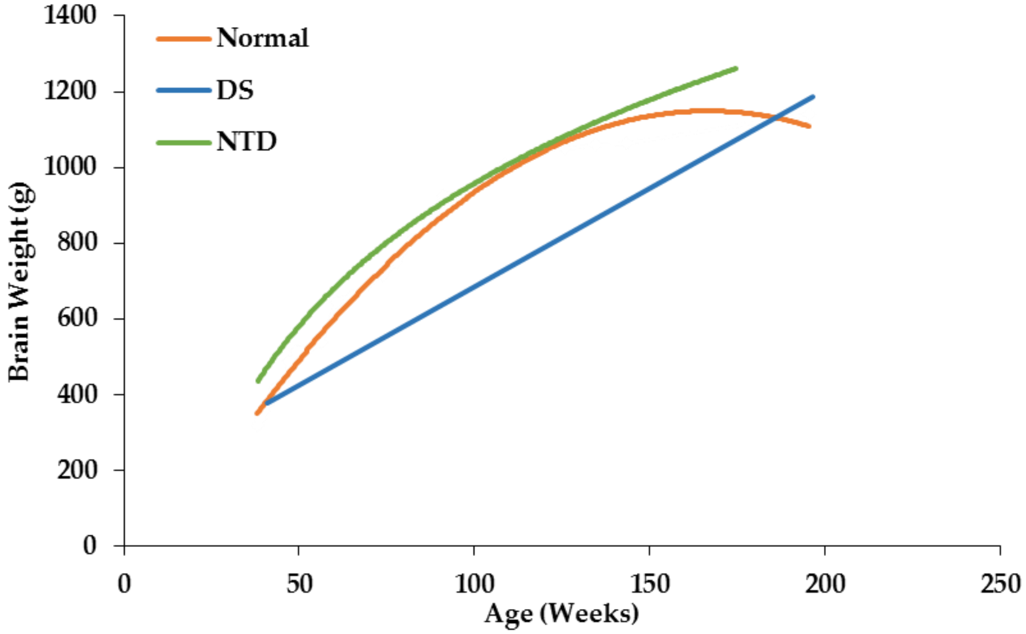

Figure S6. Comparison between brain weight and age in DS and NTD. Regression analysis showing a greater increase in brain weight with age in the NTD compared to DS cases. Note NTD growth rate was similar to the normal rate obtained from the examination of 1043 cases by Coppolleta and Wobach [76], than in DS.
